# Overexpression of a single *Leishmania major* gene enhances parasite infectivity *in vivo* and *in vitro*

**DOI:** 10.1111/j.1365-2958.2010.07130.x

**Published:** 2010-04-01

**Authors:** Linda Reiling, Mareike Chrobak, Christel Schmetz, Joachim Clos

**Affiliations:** Bernhard Nocht Institute for Tropical MedicineHamburg, Germany

## Abstract

We identified a *Leishmania major*-specific gene that can partly compensate for the loss of virulence observed for *L. major* HSP100 null mutants. The gene, encoding a 46 kD protein of unknown function and lineage, also enhances the virulence of wild type *L. major* upon overexpression. Surprisingly, the approximately sixfold overexpression of this protein also extends the host range of *L. major* to normally resistant C57BL/6 mice, causing persisting lesions in this strain, even while eliciting a strong cellular immune response. This enhanced virulence *in vivo* is mirrored *in vitro* by increased parasite burden inside bone marrow-derived macrophages. The localization of the protein in the macrophage cytoplasm suggests that it may modulate the macrophage effector mechanisms. In summary, our data show that even minor changes of gene expression in *L. major* may alter the outcome of an infection, regardless of the host's genetic predisposition.

## Introduction

*Leishmania major* is the causative agent of zoonotic, cutaneous leishmaniasis. Like all leishmaniae, these parasites appear in two morphologically distinct life cycle stages. The flagellated, cigar-shaped promastigote proliferates in the digestive tract of the vectors, phlebotomine sandflies, and infects mammals, including humans. Inside the mammalian host, the parasites develop into the non-flagellated amastigote stage that persists and proliferates inside various immune cells, such as monocytes, tissue macrophages, but also dendritic cells (Bogdan and Rollinghoff, 1999). The infiltration of immune effector cells into the infected tissue causes ulcerating, but usually self-healing skin lesions in humans.

Among the leishmaniae, *L. major* is the chosen model for experimental infections. The mouse infection models of *L. major* have given researchers a wealth of information over the last two decades, not only about this particular host-parasite interaction, but also about the general response of the mammalian immune system to invading pathogens. In particular, the striking differences observed between the course of *L. major* infections in different mouse strains (Handman *et al*., 1979) have helped to establish the antagonistic effects of cellular and humoral immune responses (Lohoff *et al*., 1998; Bogdan and Rollinghoff, 1999).

Typically, infection of C57BL/6 mice with *L. major* causes minor, transient swellings at the inoculation site that heal spontaneously within 2–3 weeks. This course of infection was correlated with an early T_H_1 type, cellular immune response, characterized by the production of specific cytokines, such as interleukin (IL)-12 and γ-interferon (IFNγ). By contrast, a cutaneous infection of BALB/c mice with *L. major* leads to a progressive, ulcerating skin lesion and heavy parasite load in the local lymphatic system. This is correlated with a T_H_2-driven, humoral immune response and characterized by increased IL-4 levels (Bogdan *et al*., 1996; Bogdan and Rollinghoff, 1998). From these findings, it was deduced that the genetic predisposition of the host animal largely determines the outcome of *L. major* experimental infections.

However, the genetic makeup of the parasite also has a strong impact on infectivity and virulence. Patient isolates of *L. infantum* were found to comprise a variety of clonal lines with varying virulence and tropism (Garin *et al*., 2001). Even the T_H_1/T_H_2 dichotomy of the early immune response may be influenced strongly by clonal variations of the infecting parasite (Reiling *et al*., 2006).

The factors governing inherent virulence of the parasite are less known. Reverse genetic analyses showed that the replacement of certain genes may impair intracellular parasite viability or parasite infectivity (Mottram *et al*., 1996; Hubel *et al*., 1997; Zhang and Matlashewski, 1997; Wiese, 1998). However, reverse genetics cannot identify unknown virulence markers as only likely candidate genes will be subjected to replacement and phenotype analysis.

The gene encoding HSP100 was established as virulence factor by a reverse genetic approach (Hubel *et al*., 1997; Krobitsch and Clos, 1999). It is expressed almost exclusively in the amastigote stage (Krobitsch *et al*., 1998; Rosenzweig *et al*., 2008). Consequently, *L. major hsp100^-/-^* gene replacement mutants which lack this gene were found to be avirulent in BALB/c mice and non-infectious to isolated macrophages while showing only minor effects in the promastigote stage. Further work both with *L. major* and *L. donovani* established a stringent requirement for HSP100 inside the host cells, but not in any axenic culture stages including *in vitro* generated amastigotes of *L. donovani* (Krobitsch and Clos, 1999).

Recently, we found that spontaneous clonal divergence within an *L. major hsp100^-/-^* population lead to the emergence of parasites with recovered virulence (Reiling *et al*., 2006). The molecular basis for this spontaneous gain of virulence is unknown. We therefore decided to use the *L. major hsp100^-/-^* mutant in a functional cloning screen to identify genes and proteins that can restore virulence to this attenuated mutant. The use of functional cloning, or complementation genetics, is well established in *Leishmania spp.* and facilitates the unbiased search for genes for selectable traits, such as drug resistance (Choudhury *et al*., 2008), but also lipophosphoglycan synthesis (Ryan *et al*., 1993b; Descoteaux *et al*., 1995; 2002;) and virulence (Beverley and Turco, 1998; Zhang and Matlashewski, 2004).

Here, we describe the identification and characterization of a virulence gene, encoding a 46 kD protein that restores infectivity to *L. major hsp100^-/-^*, boosts wild type *L. major* virulence and widens the host range while also increasing the parasite burden in macrophages.

## Results

### Preparation of a cosmid library from *L. major hsp100^-/-^*

In order to identify genes that compensate the loss of virulence of the *L. major hsp100^-/-^*, we chose a clone, not previously described, in which the HSP100 alleles are replaced by hygromycin phosphotransferase and puromycin acetyltransferase genes. This clone allows for the G418 selection of cells transfected with the cosmid vector pcosTL and its derivatives. To exclude the HSP100 gene from the screen, we prepared a cosmid library from the genomic DNA of the null-mutant and generated a recombinant population of *L. major hsp100^-/-^*[pcoslibrary].

### Functional cloning

**Fig. 1:**
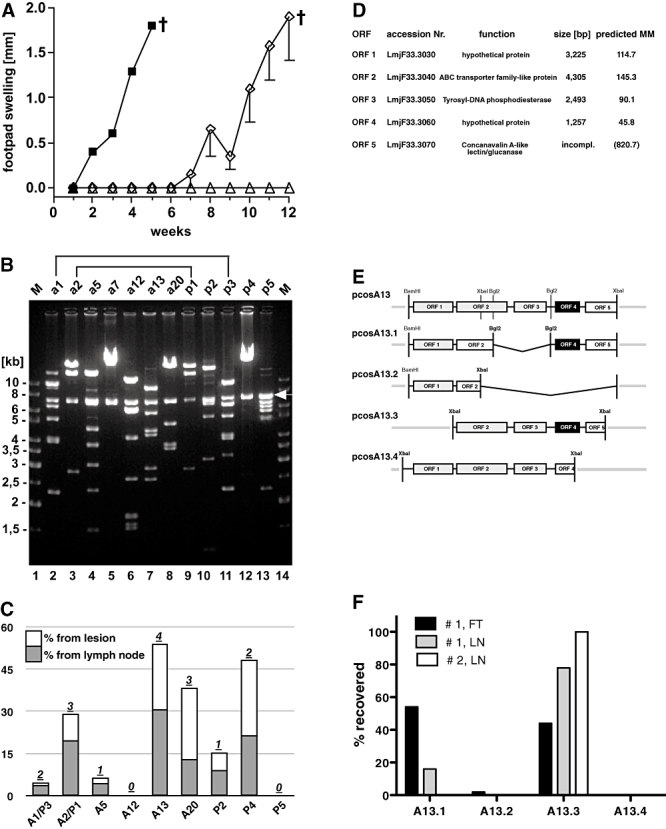
*In vivo* screening. A. Lesion formation in Balb/c mice. A total of 3 × 10^7^ stationary phase promastigotes of *L. major* wild type (solid squares), *L. major* hsp100^-/-^ transfected with pcosTL vector (open triangles) or *L. major* hsp100^-/-^[pcoslibrary] (open diamonds) were inoculated subcutaneously into the hind footpads of female Balb/c mice. Footpad swelling for each group (*n* = 4) was monitored at weekly intervals. The error bars indicate the SEM. †: time point of euthanasia. B. Characterization of selected cosmids by restriction fragment length analysis. Cosmids were cut with EcoRV and XbaI, and the DNA fragments were separated by field inversion gel electrophoresis (FIGE). Identical fragment length patterns are marked by brackets. The arrow marks the pcosTL backbone. Prefix a: *in vivo* selected cosmids; prefix p: *in vitro* selected cosmids; M: marker lane. C. Results of the secondary *in vivo* screen. *L. major* was transfected with the cosmids isolated in the primary screen (B). A representative mix of these recombinants was used for BALB/c mouse infections. Parasites were recovered from footpad lesions (white bars) and draining lymph nodes (grey bars). Distribution of the different cosmids in the selected parasites was determined by subcloning in *E. coli* (100 clones each) and clonal restriction fragment analysis (not shown). For each cosmid prototype its relative abundance (in %) is shown. Numbers on top of the bars: number of animals (out of five) from which each cosmid was recovered. D. Open reading frames (ORFs) of the cosmid pcosA13 with accession numbers, putative function, size and predicted molecular mass (MM × 1000). E. Schematic representation of pcosA13 derivatives. Cosmids pcosA13-BglI2 and pcosA13-Xba2 were constructed by digestion of pcosA13 with BglII and XbaI, respectively, and subsequent re-ligation. Cosmids pcosA13.3 and pcosA13.4 were isolated by DNA hybridization from the plated cosmid library and characterized by terminal sequencing. Shaded boxes symbolize intact ORFs; open boxes show incomplete ORFs. Grey lines represent vector DNA. ORF 4 is highlighted by inverse print. F. Frequencies of 4 pcosA13 derivatives recovered from three tissues taken from two infected animals: mouse #1 foot (#1, FT) and draining lymph node (#1, LN), and mouse #2 lymph node (#2, LN).

To select for recombinant parasites with restored virulence, the *L. major hsp100^-/-^*[pcoslibrary] population was subjected to selection by passage in BALB/c mice. *L. major* wild type and the parent null-mutant were used as controls. Figure 1A shows the course of the experimental infections. Wild type *L. major* caused rapid footpad swelling, starting at 2 weeks post infection, while no footpad swelling could be observed with the null-mutant, confirming its attenuated phenotype. Null-mutants carrying the cosmid library DNA showed an intermediate virulence. Lesions appeared 8 weeks post infection. Obviously, some of the cosmids restored virulence to the *hsp100^-/-^* mutant.

To recover the virulence promoting cosmids, the mice infected with the recombinant parasite population were sacrificed. Promastigotes were grown from lesion and lymph node tissue. Cosmid DNA from the selected populations was then isolated and cloned in *Escherichia coli*. Fifty clones each of bacteria transformed with skin- or lymph node-derived cosmids were subjected to analytical restriction fragment length analysis, using XbaI and EcoRV digestion followed by agarose gel electrophoresis (not shown). We identified 22 cosmid prototypes, named pcosA1 to pcosA22, of which seven dominated the selection and were entered into a secondary screening.

In parallel, two *L. major hsp100^-/-^*[pcoslibrary] populations were cultivated in liquid medium for 26 days. This was done to select for and exclude cosmids that confer an *in vitro* growth advantage. The cosmid DNA was isolated and cloned in *E. coli*. A total of 100 clones were also analysed by XbaI/EcoRV restriction fragment length analysis (not shown). Nine cosmid prototypes were found, named pcosP1 to pcosP9, five of which were abundant and added to the secondary screen.

We performed field inversion gel electrophoresis to compare the restriction fragment length patterns of the preselected cosmids. Two of the cosmids selected in the amastigote stage showed patterns indistinguishable from cosmids that had been favoured in the promastigote stage, namely pcosA1/pcosP3 and pcosA2/pcosP1 (Fig. 1B). We suspected that these cosmids were not favoured in the mouse but during the subsequent *in vitro* cultivation.

All preselected cosmids were subjected to partial sequence analysis of their genomic DNA inserts. By aligning the obtained sequences with the *L. major* Genome Project database, we determined which genomic regions were represented. Contrary to our expectations, none of the selected cosmids contain genes that code for molecular chaperone proteins, e.g. HSP70, HSP40 or HSP90 (Table 1). This indicates that HSP100 is a unique chaperone protein in *Leishmania* which cannot be substituted by other heat shock proteins.

**Table 1:** Cosmids selected in the screen.

Cosmid	Gene I.D.	Description
pcosA13	LmjF33.3030	hypothetical protein, conserved
	LmjF33.3040	ABC transporter family-like protein
	LmjF33.3050	Tyrosyl-DNA phosphodiesterase
	LmjF33.3060	hypothetical protein, conserved
	LmjF33.3070	Concanavalin A-like lectin/glucanase (430 of 7552 aa)
pcosA20	LmjF24.1420	lectin/glucanase
	LmjF24.1430	kinesin, putative
	LmjF24.1435	conserved hypothetical protein
	LmjF24.1440	conserved hypothetical protein
	LmjF24.1450	protein kinase, putative
	LmjF24.1460	conserved hypothetical protein
	LmjF24.1470	conserved hypothetical protein (693 of 2428aa)
pcosA2/P1	LmjF05.0710	CYC2-like cyclin, putative
	LmjF05.0720	phophatase-like protein
	LmjF05.0730	hypothetical protein, conserved
	LmjF05.0740	hypothetical protein, conserved
	LmjF05.0750	hypothetical protein, conserved
	LmjF05.0760	kinesin-like protein
	LmjF05.0770	hypothetical protein, unknown function

The putative proteins encoded by the three dominant cosmids: pcosA13, pcosA20 and pcosA2/P1. Data taken from the TritrypDB (http://tritrypdb.org/tritrypdb/).

To determine which of the cosmids provided the strongest effect on parasite survival in mice, we transfected each preselected cosmid individually into *L. major hsp100^-/-^*. The individual transfectants were mixed at equal ratios and used to infect BALB/c mice (*n* = 5). Again, wild type *L. major* and *L. major hsp100^-/-^* were used as controls. Footpad swelling was monitored weekly (not shown). As predicted, the mixed *hsp100^-/-^* population transfected with the preselected cosmids showed an intermediate virulence (not shown), confirming the results of the first selection.

Parasites were recovered and grown *in vitro* from skin lesion tissue and draining lymph nodes. Cultivation of *L. major* from the lymph node of mouse #4 failed due to bacterial contamination. Cosmids from the remaining parasite cultures were cloned, subjected to XbaI and EcoRV digestion and analysed by agarose gel electrophoresis (images not shown). The prevalence of the various cosmid types was then determined for each source (five mice, two tissues each). Figure 1C shows the results. The pcosA13 was the most prominent cosmid in the secondary selection. It was recovered from four out of five mice. In addition, it was not selected in the promastigote culture, suggesting a specific effect inside the host. Cosmid pcosA20 also showed high prevalence, but was recovered only from three out of five mice. It, too, was only favoured during the mouse passage. Cosmid pcosA2/P1 was recovered from three mice, but it was also favoured in the promastigote and thus excluded from further analysis. The remainder of the cosmids showed lower overall recoveries and were counted out. Therefore, we conclude that pcosA13 contains gene(s) that overcome the attenuated phenotype of *L. major hsp100^-/-^* in mice.

### Characterization of pcosA13

The insert of pcosA13 represents sequences from *L. major* chromosome 33, between positions 1 470 145 and 1 508 412. They comprise four complete and one partial open reading frames (Fig. 1D). LmjF33.3030 (ORF-1) encodes a hypothetical protein of 115 kD. The 156 kD product of LmjF33.3040 (ORF-2) is annotated as an ABC transporter protein, while LmjF33.3050 (ORF-3) codes for a putative tyrosyl DNA phosphodiesterase. The last intact open reading frame, LmjF33.3060 (ORF-4), is listed as encoding a 46 kD protein of unknown function. The fifth, incomplete ORF, LmjF33.3070, is part of the coding sequence for a hypothetical, conserved 821 kD polypeptide.

To determine which of the five coding sequences are responsible for the restored virulence, derivatives and variants of pcosA13 were generated either by truncation (pcosA13.1 and pcosA13.2) or by selection from the cosmid library (pcosA13.3 and pcosA13.4). These were used for a third round of selection and are shown schematically in Fig. 1E.

*Leishmania major hsp100^-/-^* was transfected with cosmids pcosA13.1-4. The transgenic populations were mixed at equal ratio and used to infect BALB/c mice. As before, we observed lesion formation with the wild type controls and with the recombinant parasite inoculates, but not with the parent *hsp100^-/-^* mutant (not shown). Parasites were isolated from the infected mice, and the distribution of pcosA13 derivatives was determined (Fig. 1F). Two cosmids, pcosA13.3 and pcosA13.1, were recovered from the surviving parasites. Only ORF 4 is common to both constructs. Conversely, cosmids pcosA13.4 and pcosA13.2 were not recovered. Both cosmids lack the ORF 4. Therefore, the third selection implicates LmjF33.3060 (ORF 4) as responsible for the restored virulence.

### Analysis of ORF 4 (LmjF33.3060)

**Fig. 2:**
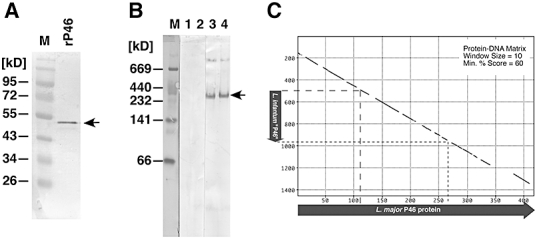
Characterization of ORF 4. A. Western blot of recombinantly expressed 46 kD protein probed with anti-P46 antiserum. Marker bands are annotated; arrow indicates the recognized 46 kD protein band. B. Native gradient gel electrophoresis and Western blot. Promastigotes of *L. major* (lanes 1, 3) or *L. major*[pcosP46] (lanes 2, 4) were lysed under non-denaturing conditions, and the lysates were separated by pore gradient gel electrophoresis. Following Western transfer, the membranes were probed either with naive mouse serum (lanes 1, 2) or with anti-P46 serum (Lanes 3, 4). The marker lane (M) blot was stained with Coomassie Brilliant Blue. Marker protein positions are indicated to the left. The arrow points at the major P46-specific band. C. Protein-DNA matrix plot (Pustell, Mac Vector™ Suite) of *L. major* P46 amino acid sequence against the corresponding region of the *L. infantum* genome (CHR 33, position 1 371 444–1 372 905). The blue arrows show the relative extent of the open reading frames; plot parameters are given in the top right corner. Extent of the *L. infantum* open reading frame relative to the *L. major* sequence in indicated (dotted lines).

The LmjF33.3060 gene encodes a 46 kD protein. Using recombinantly expressed 46 kD protein (P46), we immunized mice and obtained antisera which recognize the antigen faithfully in a Western blot (Fig. 2A). We also performed a native gradient gel electrophoresis to assess the native size of P46 (Fig. 2B). While naive mouse sera do not detact bands in the blot, the anti-P46 antisera decorate a protein band corresponding to a native molecular mass of ∼300 000, possibly representing a hexameric complex.

We performed extensive database mining to identify putative functional domains and motifs within P46 and to identify related genes/proteins. The amino acid sequence does not contain known functional motifs or localization signals. A search for homologues turned up only one homologue, a partially conserved putative gene in *L. infantum*. A protein to DNA matrix plot (Fig. 2C) shows conservation between the *L. major* protein and the cognate *L. infantum* genomic region. However, the *L. infantum* sequence contains only a small open reading frame representing less than 40% of the *L. major* amino acid sequence. A similarly incomplete conservation was found when we compared P46 with the preliminary sequence (http://www.sanger.ac.uk/sequencing/Leishmania/mexicana/) from the corresponding region of *L. mexicana* chromosome 32 (not shown). No open reading frame was found in the corresponding genomic region of *L. braziliensis.* Therefore, P46 appears to be unique to *L. major*.

### Overexpression of P46 enhances virulence in BALB/c mice

To verify P46 as the virulence enhancing gene product, we constructed an episome that contains only LmjF33.3060 plus its 5′- and 3′ untranslated flanking regions. The resulting cosmid, pcosP46, and the vector, pcosTL, were both transfected into *L. major* and into *L. major hsp100^-/-^*, to test the effect of P46 overexpression in both genetic backgrounds. The recombinant strains were grown in liquid medium and their proliferation rates were indistinguishable from wild type *L. major* (data not shown).

**Fig. 3:**
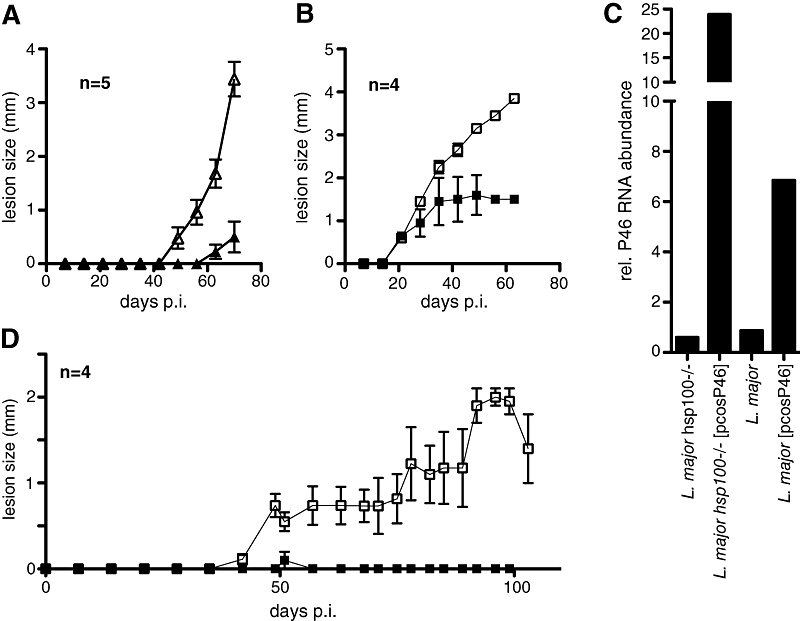
P46 Overexpressing Parasites in the Mouse Infection Model. A. *L. major hsp100-/-* (solid triangles) or *L. major hsp100-/-*[pcosP46] (open triangles) were inoculated into footpads of BALB/c mice (*n* = 5). Footpad swelling [mm] was monitored at weekly intervals. B. Infection of BALB/c mice (*n* = 4) with *L. major* (solid squares) or *L. major*[pcosP46] (open squares). Footpad swelling was monitored weekly. C. Real-time RT-PCR. The relative P46-specific mRNA levels in log phase promastigotes of *L. major hsp100^-/-^*, *L. major hsp100^-/-^*[pcosp46], *L. major* wild type and *L. major*[pcosP46] were determined by whole cell RNA extraction, cDNA synthesis and subsequent P46-specific real-time PCR. Results are given as multiples of *L. major* wild type expression levels. D. C57BL/6 mice (*n* = 4) were infected with either *L. major* (solid squares) or *L. major*[pcosP46] (open squares). Footpad swelling was monitored over 110 days.

The P46 overexpressing parasites were tested in the mouse infection model. As expected, overexpression of P46 in *L. major hsp100^-/-^* restored virulence to the mutant. Detectable lesions appeared 7 weeks post infection (Fig. 3A), while the parent null-mutant did not cause lesions within the observation span, except for one animal where a lesion developed at 9 weeks. This confirms that P46 is indeed responsible for the partial reversal of the *hsp100^-/-^* phenotype.

Overexpression of P46 also had significant effects on wild type *L. major* virulence. While both *L. major* and *L. major*[pcosP46] induced lesions within 3 weeks, the lesions caused by the latter progressed further (Fig. 3B). Even at these extreme lesion sizes, we did not observe a tendency towards ulceration, indicating an impact of P46 on the interaction of the parasite with the host immune system.

This result shows that the P46 overexpression is not a functional complementation for the lacking HSP100. Rather, P46 enhances virulence and thus compensates for the attenuated phenotype. Although unexpected, the strong effect of P46 overexpression on the virulence of wild type *L. major* was deemed sufficiently important and worthwhile of further investigation.

We ascertained P46 overexpression by real-time RT-PCR of RNA isolated from logarithmic growth phase promastigotes, using actin mRNA as internal standard. P46 expression in wild type *L. major* is elevated by a factor of 6 due to the pcosP46 transgene, compared with a 24-fold overabundance in the *hsp100^-/-^* mutants carrying the cosmid (Fig. 3C). Of note is that the recombinant parasites had already undergone one mouse passage at the point of the analysis. Therefore, in the hsp100^-/-^ mutant, the selective pressure on P46 overexpression was probably higher, due to the low inherent virulence of this mutant.

### P46 overexpression boosts *L. major* virulence in C57BL/6 mice

The observations in the BALB/c mouse model led us to test the effects of P46 overexpression in the C57BL/6 mouse model. In these mice, *L. major*-induced lesions are minor and transient. Indeed, we observed a minor swelling at 5 weeks post infection with *L. major* wild type (Fig. 3D, solid squares). By contrast, *L. major*[pcosP46] causes prominent and lasting lesions in C57BL/6 mice (Fig. 3D, open squares). The observed footpad swelling correlates with increased persistence and/or proliferation of the P46 overexpressing parasites which we could detect by histological staining and microscopy (data not shown). Nevertheless, the infection was controlled at a moderate lesion size and towards the end of the observation time the footpad swelling began to recede. Obviously, C57BL/6 mice are ultimately able to control the infection. Therefore, P46 overexpressing parasites can temporarily overcome or outgrow the protective immune response in the early stages of infection in C57BL/6 mice.

### P46 does not suppress the T_H_1 immune response of C57BL/6 mice

**Fig. 4:**
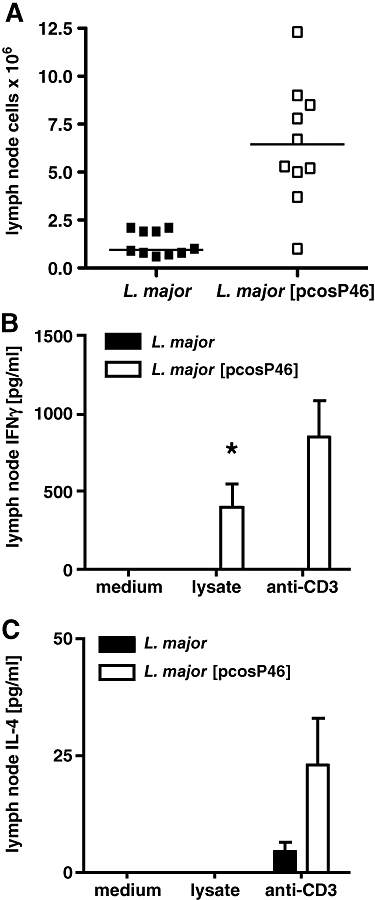
Cytokine response in C57BL/6 mice. C57BL/6 mice were infected with *L. major* or *L. major*[pcosP46]. At day 17 post-infection draining lymph nodes were isolated. Lymph node cells were counted (A) and triplet samples of 3 × 10^5^ cells each were stimulated with anti-CD3 or *L. major* soluble antigen. After 72 h, the supernatants were collected and analysed by sandwich ELISA for either IFNγ (B) or IL-4 (C). The data are the results of one experiment with 10 mice per group. Values are given as means (± SEM). Asterisk: *P* < 0.05.

Resistance to *L. major* infection is normally correlated with an early induction of the T_H_1 arm of the immune response. A hallmark of the T_H_1 type response is the enhanced production of IFNγ and the repression of IL-4 synthesis. Faced with the persistance of P46 overexpressing *L. major* in C57BL/6 mice, we tested cytokine production in draining lymph nodes 17 days post infection, using IL-4 and IFNγ sandwich ELISA. First, we recovered a threefold higher number of cells from the lymph nodes of mice infected with *L. major*[pcosP46], compared with control infections (Fig. 4A). Thus, infection with P46 overexpressing *L. major* causes an increased influx of cells into the draining lymph nodes. We did not observe IFNγ production in the mice infected with *L. major* wild type (Fig. 4B). By contrast, *L. major*[pcosP46] induced strong IFNγ production either with *L. major* lysate or with anti-CD3 antibody (Fig. 4B). IL-4 production was weakly induced by anti-CD3 challenge, but not using *Leishmania* lysate, with *L. major*[pcosP46] infection causing a slightly stronger response (Fig. 4C). These results demonstrate that C57BL/6 mice still mount a strong, potentially protective T_H_1 type immune response against the P46 overexpressing *L. major*. Therefore, the persistence and proliferation of *L. major*[pcosP46] does not suppress the predominant T_H_1 immune response typical for C57BL/6 mice.

We also performed equivalent experiments in BALB/c mice which typically mount an IL-4-driven T_H_2 immune response after *L. major* infection. Here, we observed no significant differences in cytokine production between mice infected with wild type or P46 overexpressing parasites (data not shown). Hence, the cytokine expression pattern in mice does not change in response to P46 overexpression in *L. major*. This result hints at an impact of P46 on host defence mechanisms downstream of T helper cell activation.

### Localization of P46 in *L. major* promastigotes

**Fig. 5:**
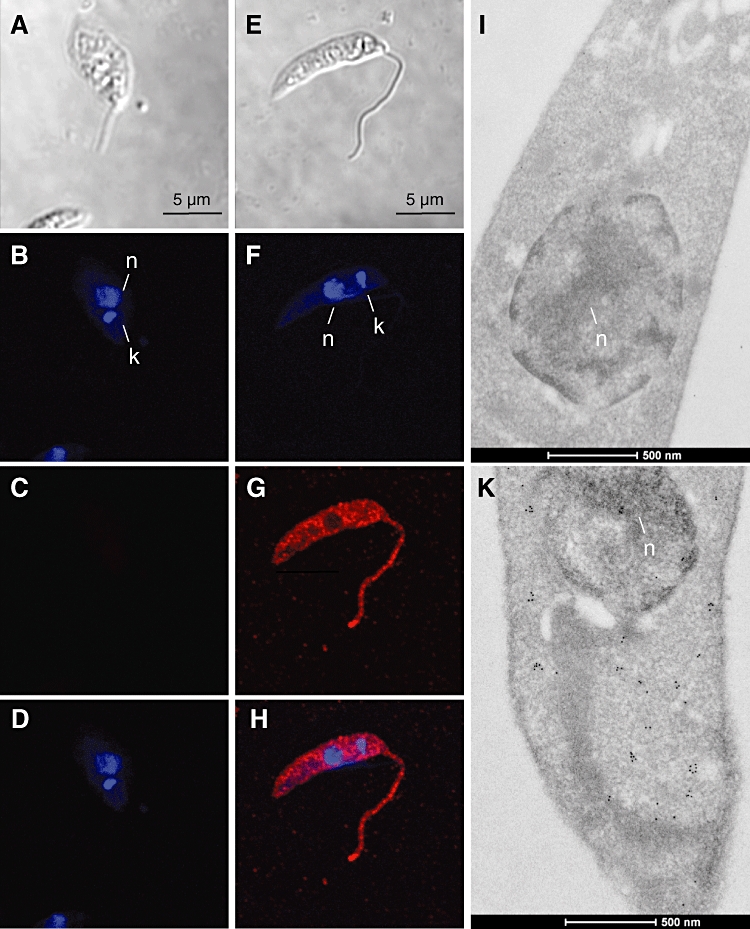
P46 localization in promastigotes. (A–H) Indirect immunofluorescence. *L. major* promastigotes were probed without primary antibody as controls (A–D) or with anti-P46 antiserum (E–H). Phase contrast (A,E); DAPI staining of nucleus and kinetoplast (B,F). Immune detection without (C) or with (G) anti-P46 serum. D and H show overlays of immune detection and DAPI stain (I–K). Immunogold (10 nm) electron microscopy of *L. major*[pcosP46] promastigotes using naive mouse serum (I) or anti-P46 serum (K). n = nucleus. 500 nm size bar at the bottom.

The surface molecules of *L. major* have been shown to impact on the infectivity of the parasites. To test for the possibility that P46 might also be found on the surface of *L. major*, we first determined its localization in promastigotes, using immune fluorescence microscopy. The antibody decorates the cytoplasm, including the flagellum, and sparing both nucleus and kinetoplast (Fig. 5E–H). There is no evidence for a surface localization. Identical results were obtained with a second batch of antiserum from a different animal (not shown). No fluorescence is obtained using secondary antibodies alone (Fig. 5A–D) or in conjunction with pre-immune mouse serum (not shown).

To confirm this cytoplasmic localization, we also performed immune electron microscopy. While naive mouse sera fail to decorate the promastigote microsections (Fig. 5I), the P46 antiserum recognizes distinct foci in the cytoplasm of the parasites (Fig. 5K). The clustered appearance hints at the formation of multimeric complexes and supports the results from the native electrophoresis (Fig. 2B). We conclude that P46 is a cytoplasmic protein in promastigotes, localizing to distinct foci.

### Localization of P46 within the infected macrophage

**Fig. 6:**
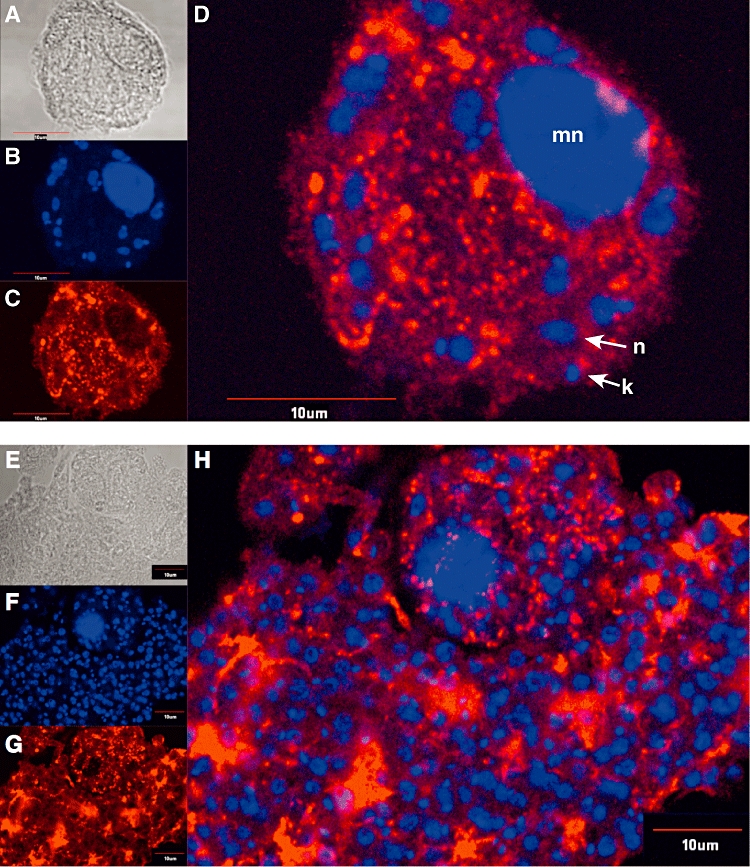
P46 localization in infected macrophages. Bone marrow-derived macrophages were infected with *L. major* (A–D) or *L. major*[pcosP46] (E–H) at a 1:10 cell ratio and stained with anti-P46 antiserum and DAPI. Bright field images (A,E), DAPI stain (B,F) and anti-P46 stain (C,G) are shown. (D,H): DAPI and anti-P46 overlays. mn = macrophage nucleus; k = kinetoplast; n = parasite nucleus. 10 µm size bars are shown.

Because P46 exerts its virulence enhancing effects during the mammalian stage of the parasite's life cycle, we next analysed the distribution of P46 in amastigotes within infected macrophages. To this end, bone marrow-derived macrophages were infected with either wild type *L. major* or with *L. major*[pcosP46]. After 24 h, infected cells were fixed and stained with anti-P46 serum and DAPI. No anti-P46 staining is observed in uninfected macrophages (data not shown). Figure 6A–D shows the result for the infection with *L. major* wild type. The DAPI stain marks the macrophage nucleus (mn), but also the parasite nucleus (n) and kinetoplast (k). The anti-P46 staining is found in distinct foci within the macrophage cytoplasm and does not match the localization of the parasites. This is even more pronounced in macrophages infected with the *L. major*[pcosP46] (Fig. 6E–H). The P46 foci in these macrophages are larger and found within clusters, often in small areas that are free of parasites.

**Fig. 7:**
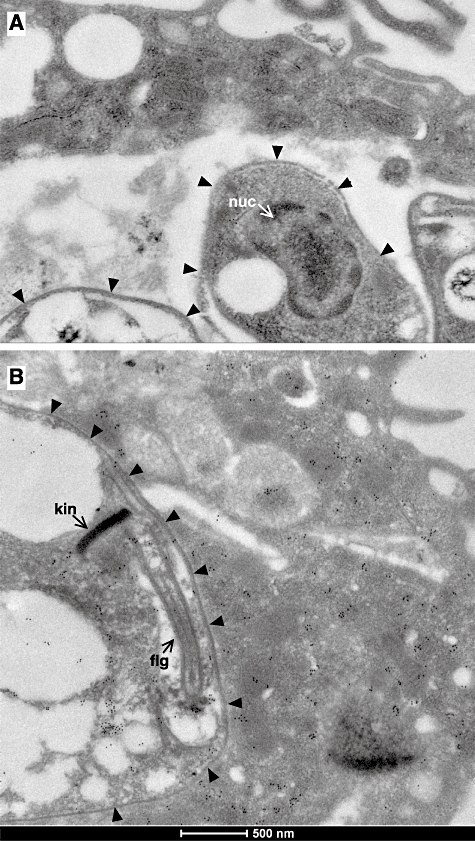
Immune electron microscopy of infected macrophages. Bone marrow-derived macrophages were infected with *L. major*[pcosP46] at a 1:10 cell ratio. After 24 h, infected cells were suspended and subjected to immunogold (10 nm) electron microscopy, using naive mouse serum (A) or anti-P46 serum (B). Arrow heads delineate the parasitophorous vacuole membranes, 500 nm size bars are shown at the bottom. kin = kinetoplast, flg = flagellum, nuc = nucleus.

A co-staining, using anti-lamp1 sera to highlight endosomes, showed no significant colocalization with the anti-P46 staining (data not shown), arguing against a tight association with the parasitophorous vacuoles. To clarify this, we performed an immune electron microscopy of *L. major*[pcosP46]-infected bone marrow-derived macrophages. Again, naive mouse sera do not stain the infected macrophage or the intracellular parasites (Fig. 7A). By contrast, the anti-P46 antibodies decorate focal assemblies of antigen both inside the parasite and in the cytoplasm of the infected macrophage (Fig. 7B). This result confirms that P46 is exported to the macrophage and shows that the protein is not associated with the endosome compartment. The presence of P46 inside the parasites was not detected by indirect immunofluorescence. This may be due to inefficient diffusion of antibody through host cell cytoplasmic membrane, parasitophorous vacuole membrane and parasite cytoplasmic membrane, but it may also be owed to the superior resolution of immune EM.

### P46 boosts infectivity *in vitro*

**Fig. 8:**
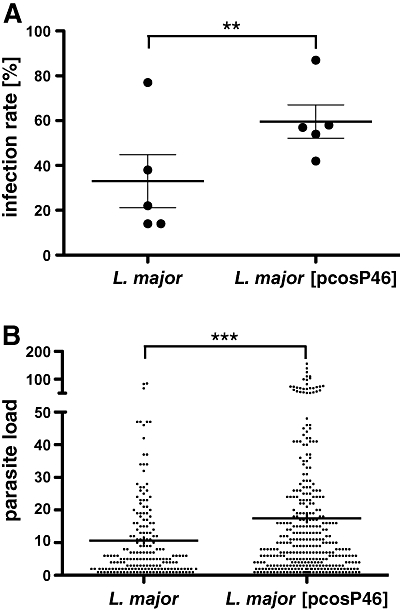
*In vitro* macrophage infection experiments. Bone marrow-derived macrophages were infected with *L. major* or *L. major*[pcosP46] at a 1:10 cell ratio. Macrophages (100–200 per sample) were analysed for parasite infection. The infection rate as percentage of infected macrophages, and the parasite load as number of parasites per infected macrophage were determined by fluorescence microscopy. A. Scatter plot of infection rates (*n* = 5). Significance was tested by paired *t*-test; *P* (two-tailed) = 0.001. B. Scatter plot of parasite loads (*n* = 600). *P* (two-tailed) = 5.9 E-06, *U*-test.

The indirect immune fluorescence images in Fig. 6 also suggested a greater parasite load for macrophages infected with *L. major*[pcosP46]. This prompted us to determine infection rates and parasite loads after *in vitro* infection. For this, we performed a series of five infection experiments with *L. major* wild type and *L. major*[pcosP46] in bone marrow-derived macrophages. For each experiment, 100–200 macrophages were viewed. Infection rates and parasite loads were quantified by fluorescence microscopy after DAPI staining. Infection rates of macrophages increased significantly (*P* = 0.01) from 33% to 59.8% due to P46 overexpression (Fig. 8A). Also, average parasite loads (Fig. 8B) defined as amastigotes per infected macrophage increased almost twofold (10.7–17.5). This difference is highly significant (*P* < 0.0001). Both effects add up to threefold increase of parasite burden in the cultured macrophages. We conclude that P46 overexpression confers a substantial advantage to *L. major* inside mammalian macrophages, explaining and confirming the *in vivo* infection data.

## Discussion

Genetic complementation is a tried strategy in *Leishmania spp.* and has been used to identify a variety of genes responsible for a diverse set of traits (Ryan *et al*., 1993a,b; Turco *et al*., 1994; Beverley and Turco, 1995; Descoteaux *et al*., 2002; Coelho *et al*., 2003; Choudhury *et al*., 2008). Episomes are maintained stably in *L. major*, and even in the absence of antibiotic selection, such transgenes can persist for multiple generations in the animal host (Hubel *et al*., 1997; Hoyer *et al*., 2004). The foremost advantage of functional cloning is the potential to select genes according to their function, regardless of preconceptions. The unbiased nature of complementation genetics makes it likely to identify heretofore uncharacterized genes and proteins and implicate them in cellular functions and structures.

Using an attenuated *L. major* clone, *L. major hsp100^-/-^*, we have screened a genomic DNA library for genes that may reverse that attenuation, i.e. restore parasite virulence. Indeed, we were able to isolate a small number of cosmids that fulfilled this criterion. It is telling that none of the selected cosmids harbours any of the leishmanial heat shock genes. In the yeast model *Saccharomyces cerevisiae*, the loss of the HSP100 orthologue, HSP104, can be complemented in part by overexpression of HSP70 (Sanchez *et al*., 1993). There are several members of the HSP70 gene family in the *L. major* genome, but none of these genes were selected to compensate for the lack of HSP100. This underscores that induction of thermotolerance, one of the best characterized functions of yeast HSP104, may not be the key role for *Leishmania* HSP100 (Krobitsch and Clos, 1999).

Some of the selected cosmids were also favoured in *in vitro* promastigote culture and therefore excluded from consideration. Of the remaining three candidates, one dominated the *in vivo* selections and was further analysed. The virulence enhancing property could be attributed to a single gene, LmjF33.3060, which encodes a 46 kD protein of unknown function and phylogenetic lineage.

Overexpression of this protein, dubbed P46, reliably restored virulence to the hsp100^-/-^ null-mutant. Surprisingly, the effect of P46 overexpression is not confined to the null-mutant, but is also observed in a wild type genetic background. This is indication that P46 overexpression does not functionally complement the null-mutant but rather compensates for the attenuated phenotype. Thus, the approach did not further our knowledge of HSP100 function in *L. major*. In spite of this, P46 is worthwhile of attention.

There is no structural similarity to other proteins in the database, and even sequence conservation with *L. infantum* is comparatively weak. No open reading frame was found in the corresponding genomic region of *L. braziliensis.* There is no evidence of divergence for this gene within the *L. major* species, though. *L. major* Friedlin strain (Genome Project) and *L. major* 5ASKH (our study) were isolated from different geographical areas, Israel and Turkmenistan respectively (Al-Jawabreh *et al*., 2008). Yet, we find no sequence divergence between P46 genes of the two strains (data not shown). Therefore, P46 appears to be specific to *L. major* and may even serve as a molecular marker to distinguish Old World *Leishmania* species. This requires further analyses of various cutanotropic Old World leishmaniae.

It has been shown before that spontaneous genetic rearrangements can revert the phenotype of genetically attenuated *L. major* (Spath *et al*., 2004; Reiling *et al*., 2006). Also in the *L. major* model, it could be shown that two established laboratory strains, *L. major* Friedlin and *L. major* MHOM/IL/81/FEBNI, both of Middle Eastern origin, display different infection courses in mice deficient in TNF production (Ritter *et al*., 2004), indicating that TNF-dependent control of *L. major* infection is dependent on the parasite strain. Similarly, earlier work (Garin *et al*., 2001) showed that various clonal lines within an infecting population of *L. infantum* displayed different virulence and tissue tropism. Thus, spontaneous clonal variations may influence the outcome of an infection, compounding issues such as the host's immunological predisposition.

However, the molecular bases for these events remain unknown. Here, we describe for the first time that the relatively minor overexpression of a single gene can restore virulence and even alter the host range. The fivefold P46 overexpression in *L. major* exacerbates the pathology in BALB/c mice and allows productive and lasting infection of normally resistant C57BL/6 mice. Contrary to our expectation, this is not brought about by a reversal of the T_H_1/T_H_2 dichotomy in the C57BL/6 mice. Rather, the persisting infection with *L. major*[pcosP46] causes an increased IFNγ release. The fact that the overexpressing *L. major* can survive in the face of a strong IFNγ response indicates that P46 protects against effector mechanisms downstream of a macrophage activation. Similar results were obtained earlier with spontaneous escape variants of *L. major* hsp100^-/-^ (Reiling *et al*., 2006). As *L. major* has the ability to induce or prevent the expression of distinct host genes (Racoosin and Beverley, 1997), it should be interesting to perform an RNA microarray analysis of *L. major*-infected macrophages with and without P46 overexpression. The results may shed light on immune modulatory mechanisms of *Leishmania spp.*

This idea is supported by the results of the *in vitro* infections. Elevated P46 levels corresponded with elevated infectivity and parasite loads. Both effects combine to a tripled overall parasite load in the macrophage culture. Preliminary results show that this is not due to more efficient uptake by the macrophage, but rather through improved intracellular survival and/or proliferation (M.C., unpubl. obs.). These *in vitro* observations match and explain the results obtained in the mouse model. The strongly increased parasite numbers in the macrophage population most likely cause expansion of the infected cells and also a stronger influx of immune cells, explaining both the increased tissue swelling and the increased IFNγ stimulation.

How does P46 enhance intracellular survival/proliferation? So far, repeated attempts to generate P46 null mutants by homologous recombination failed. Neither single allele nor double allele gene replacement mutants could be raised under antibiotic selection. There are two explanations for this: (i) the expression of selection marker genes as directed by P46 untranslated flanking regions is too low for sufficient antibiotic resistance, or (ii) the expression of P46 is essential for efficient parasite growth. Our inability to even generate single allele replacements argues in favour of the former explanation. We have therefore halted these efforts.

In promastigotes, P46 shows a cytoplasmic distribution which probably changes due to stage conversion. This requires either stage specific processing or amastigote-specific export pathways. There are no conserved signal sequences in P46. However, recent work (Silverman *et al*., 2008) suggests that *Leishmania* utilizes non-classical secretion pathways dispensing with the need for signal sequences.

Indeed, new research results confirm exosomal transport as a means for protein export from Leishmania parasites to their environment (Silverman *et al*., 2010). The payload of these exosomes comprises most chaperones, including HSP100. Additional research using *L. major* should clarify whether P46 is a part of the proteins exported via exosomal pathways.

The lack of an *in vitro* stage conversion system so far precludes a kinetic study of P46 trafficking during differentiation. This is compounded by the fact that, for reasons still unknown, a P46::eGFP chimeric protein was expressed poorly in infected macrophages (data not shown). Different labelling of P46 for life imaging or the development of an axenic amastigote model for *L. major* are required to address this question.

Clearly, more information is needed about the role and function of this protein. The phylogenetic distribution of P46 among cutanotropic Old World leishmaniae and its relative levels of expression in various *L. major* isolates should be researched, to establish possible correlations with geographical or clinical parameters. New attempts to generate P46 null mutants must be started, using variations of standard protocols. Lastly, macrophage gene expression should be analysed to detect the impact of P46 overexpression on leishmanicidal or immune modulatory mechanisms.

## Experimental procedures

### Parasite culture

Promastigote *L. major* (MHOM/SU/73/5ASKH) were grown at 25°C in modified Medium199 (Sigma-Aldrich, with 20% heat inactivated FCS, 40 mM HEPES pH 7.4, 0.2% NaHCO_3_, 100 µM adenin, 1.2 µg ml^−1^ 6-biopterin, 10 µg ml^−1^ haem, 20 µg ml^−1^ gentamicin, 2 mM l-glutamin, pH 7.0). For macrophage and mouse infection experiments, parasites were allowed to grow to stationary phase. Promastigotes were counted using a CASY cell counter (Roche).

### Generating *L. major hsp100^-/-^* (Puro^r^/Hygro^r^)

The *L. major hsp100^-/-^* mutant was created by replacing the HSP100 coding sequence with neoR and hygR marker genes (Hubel *et al*., 1997). The cosmid vector pcosTL confers neomycin (G418) resistance and is not compatible with G418 resistant host strains. Therefore, in order to use a pcosTL-based library in the hsp100^-/-^ mutants, we had to replace the neomycin resistance marker from the null mutants. For this purpose, the plasmid p*L.m.clpB* hygro (Hubel *et al*., 1997) was cleaved using KpnI and BamHI, deleting the hygromycin resistance marker. Then, the puromycin resistance gene (PAC) was inserted in the same place, resulting in the plasmid p*L.m.clpB* puro. XbaI digest of this p*L.m.clpB* puro plasmid resulted in a fragment encomapssing the *HSP100* untranslated regions flanking the PAC gene. This fragment was used to transfect *L. major hsp100^-/-^* (Neo^r^/Hygro^r^), to target the neomycin phosphotransferase gene in the HSP100 gene locus for replacement. Clonal selection under puromycin and hygromycin selected for parasites that had replaced the neomycin phosphotransferase gene against the PAC gene. A clone (TF3.1) resistant to puromycin but not neomycin was verified by PCR analysis and chosen for transfection with the cosmid library.

### Preparation and screening of cosmid library

The cosmid library was generated as described previously (Choudhury *et al*., 2008), with the exception that genomic DNA of a *L. major* HSP100 null mutant was used. To prepare cosmids for transfection of *L. major*, cosmids were purified from *E. coli* by alkaline lysis, phenol/chloroform/isoamylalcohol extraction and subsequent isopropanol precipitation. Hybridization screening of the cosmid library was performed following established protocols (Sambrook *et al*., 1989) and using digoxigenin-labelled DNA probes.

### Transfection

The transfection procedure was performed as described (Choudhury *et al*., 2008). Briefly, parasites from logarithmic growth phase were washed twice in pre-chilled PBS (pH 7.0), once in pre-chilled electroporation buffer (21 mM HEPES pH 7.5, 137 mM NaCl, 5 mM KCl, 0.7 mM Na_2_HPO_4_, 6 mM Glucose) and resuspended in electroporation buffer to a final concentration of 1 × 10^8^ ml^−1^. An equivalent of 4 × 10^7^ parasites was mixed with 2 µg of DNA for homologous recombination, or 50 µg of DNA for stable episomal transfection. Cells were transferred to an electroporation cuvette (0.4 cm gap, Biozym) and pulsed three times using a Gene Pulser™ (Bio-Rad) at 3000 V cm^−1^ for homologous recombination, or 3750 V cm^−1^ for episomal transfection (25 µF, 200 Ω). Following electroporation, parasites were transferred to fresh, modified Medium199 and grown for 24 h without selection before puromycin (45 µg ml^−1^), hygromycin (50 µg ml^−1^) and/or G418 (neomycin) (50 µg ml^−1^) were added.

### Experimental infections

Three days prior to infection, parasites were grown in modified Medium199 until stationary phase was reached. Parasites were washed twice and resuspended in pre-chilled phosphate buffered saline pH 7.0 (PBS). Parasite suspension (1–4 × 10^7^ in 50 µl of PBS) was inoculated s.c. into the rear foot pads of BALB/c or C57BL/6 mice. Footpad size was monitored weekly. Mice were sacrificed before lesions ulcerated. For parasite reisolation, the lesion tissues and the draining lymph nodes were prepared, homogenized and suspended in modified Medium199 and the appropriate antibiotic pressure was applied. The medium was also supplemented with penicillin/streptomycin (100× PenStrep, Sigma) to avoid bacterial contamination. For cytokine quantification experiments, mice were sacrificed 17 days post infection.

### Isolation and cloning of cosmid DNA from *Leishmania*

Isolation and cloning of cosmid DNA from *Leishmania* promastigotes was performed as described (Choudhury *et al*., 2008).

### Truncated variants of pcosA13

Both ends of the cosmid insert were amplified enzymatically using specific primers. The amplification products were labelled with digoxigenin-dUTP and used to screen colony lifts on cellulose nitrate filters by hybridization. Two out of 10 cosmid clones were identified as sufficiently different from pcosA13 by terminal sequencing of the inserts (pcosA13.3; pcosA13.4). The truncated variants pcosA13.1 and pcosA13.2 were obtained by digestion with BglII or XbaI respectively. Digestion of pcosA13 with BglII and subsequent re-ligation of the truncated cosmid deleted ORF-3 and the 3′ half of ORF-2. Digestion of pcosA13 with XbaI and subsequent ligation deleted ORF-3, ORF-4, and the incomplete ORF-5, as well as 60% of ORF-2. For expression of ORF 4 alone, a ∼5400 nt segment of pcosA13 was amplified, spanning the entire 5′- and 3′-non-coding regions, and including LmjF33.3060. The primers used added XbaI sites to the amplification product, which was then ligated into XbaI-digested pcosTL vector (Kelly *et al*., 1994). The resulting cosmid, pcosP46, contains LmjF33.3060 with its natural flanking sequences.

### *In vitro* infection experiments

Murine bone marrow macrophages (BMM) were isolated from the femurs of female C57BL/6 mice and differentiated in Iscove's Modified Dulbecco's Medium (IMDM) supplemented with 10% heat inactivated FCS, 5% horse serum and 30% L929 supernatant (modified after Racoosin and Swanson, 1989). For infection, BMM were harvested, washed and seeded into the wells of an eight-well chamber slide (NUNC) at a density of 4 × 10^5^ cells per well. The macrophages were incubated for 48 h at 37°C and 9% CO_2_ to allow adhesion of the cells. Adherent BMM were infected with stationary phase promastigotes (Racoosin and Beverley, 1997) at a multiplicity of infection of 10 parasites per macrophage. After 4 h of incubation at 37°C in modified Medium199, non-phagocytosed parasites were removed by multiple washing steps with PBS and incubation was continued for another 24 h in IMDM at 37°C and 9% CO_2_. The medium supernatants was removed. The cells were washed twice and fixed in ice-cold methanol. Intracellular parasites were quantified by nuclear staining with DAPI (1.25 µg ml^−1^, Sigma) and fluorescence microscopy.

### Field inversion gel electrophoresis (FIGE)

1% agarose gels were prepared and run in fresh 0.5× TBE buffer using the following multi-phase program: Program 1: 200V, run in time 10 min; run time 8 h; pulse time 1–20 s; Mode reverse; F/R ratio 3:1. Program 2: 200V; run time 8 h; pulse time 0.8–1.5 s; Mode reverse; F/R ratio 3:1. After the run, gels were stained in ethidium bromide (10 µg ml^−1^ in 0.5× TBE buffer).

### Analysis of cytokine production

Draining lymph nodes and spleens from infected C57BL/6 or BALB/c mice were isolated 17 days post infection. After determining organ weight and cell numbers, single-cell suspensions were seeded in triplicate into 96-well plates (3 × 10^5^ cells per well), using RPMI medium supplemented with 10% heat-inactivated fetal-calf serum, l-glutamine (2 mM) and Pen/Strep (1×, Sigma). Cells were stimulated with either 6 µg ml^−1^ anti-CD3 mab or *L. major* lysate. After 72 h, supernatants were collected and preserved at −20°C. Production of interferon-γ (IFNγ) and IL-4 was assessed in a specific two-sided ELISA using supernatant of stimulated lymph node cells. Antibody pairs and cytokine standards were purchased from R&D Systems GmbH (Wiesbaden).

### Antibodies

The coding region for P46 was amplified and expressed in *E. coli* using the expression vector pJC45 as described (Schlueter *et al*., 2000). The His-tagged protein was purified from bacterial lysates by nickel affinity chromatography. The recombinant protein was used together with Freund's complete adjuvant to immunize NMRI mice, followed by two boosts with incomplete adjuvant. Heparanised blood from immunized animals was collected by cardiac puncture after CO_2_ euthanasia. Serum was obtained after centrifugation and tested by Western blotting. Anti-mouse IgG alkaline phosphatase was purchased from Dianova, Alexa Fluor 594 goat anti-mouse IgG from Invitrogen.

### SDS-PAGE and Western blotting

Production of SDS cell lysates, discontinous SDS-PAGE and Western blot were performed according to standard protocols, using FluoroTrans PVDF membranes (Pall). Membranes were blocked (5% milk powder in Tris-buffered saline, 0.1% Tween 20), probed with the polyclonal antiserum (1:250 in blocking solution) before incubation with an anti-mouse IgG-alkaline phosphatase conjugate (1:2500, Dianova). Blots were developed using nitro blue tetrazolium chloride and 5-bromo-4-chloro-3-indolyl phosphate (Sigma).

### Pore gradient gel electrophoresis

Extraction of non-denatured *Leishmania* proteins and native gradient gel electrophoresis was performed largely according to Westwood *et al*. (1991). Promastigotes (1 × 10^7^) were harvested by centrifugation, washed twice with PBS and resuspended in 40 µl of extraction buffer (15% glycerol, 0.5 mM 1,10-phenanthroline, 10 mM Tris-HCl pH 8,0, 70 mM KCl). After four freeze and thaw cycles, cell lysates were cleared by centrifugation. The supernatant, containing the soluble protein fraction, was mixed (5:1, v/v) with loading buffer (50% glycerol, 0,1% bromphenol blue). The samples were run alongside a high molecular weight protein marker for native gels (Amersham) on a 4–18% polyacrylamide (2.5–6% glycerol) gradient gel in 0.5 × TBE buffer. Electrophoresis was allowed to proceed for 24 h at 4°C, at 20 V cm^−1^. This long duration of electrophoresis was necessary for all proteins to migrate to their exclusion limit regardless of their net charge (Andersson *et al*., 1972). After that, the gel was incubated at 60°C in transfer buffer (48 mM Tris, 39 mM glycine, 0.5% SDS, 10 mM DTT) for 30 min, followed by Western blot analysis.

### Immunofluorescence and confocal microscopy

For immunofluorescence labelling, log-phase promastigotes (1 × 10^7^ cells) were washed twice and resuspended in PBS. Cells (2 × 10^5^) were immobilized on poly (l-lysine) coated microscopic slides and fixed for 10 min in acetone. Non-adherent cells were removed by a gentle wash (0.1% Triton X-100 in PBS). Slides with fixed parasites or infected macrophages were then incubated for 15 min at RT with permeabilization buffer (50 mM NH_4_Cl and 0.1% Triton X-100 in PBS), followed by a 15 min incubation in blocking solution (2% BSA and 0.1% Triton X-100 in PBS). Cells were washed thrice and incubated sequentially with: (i) dilutions of primary antibody (1:500), (ii) fluorescent secondary antibody (1:2500), (iii) with DAPI (1.25 µg ml^−1^, Sigma). After washing (3×), Mowiol (CalBiochem) and coverslips were applied. Fluorescence images were captured using an Olympus FluoView FV1000 confocal microscope and the Olympus Fluoview Ver. 1.7b software.

Immune electron microscopy was performed following established protocols (Krobitsch *et al*., 1998). *L. major* promastigotes or infected macrophages were harvested by gentle centrifugation, washed twice in Dulbecco's phosphate-buffered saline solution and fixed for 24 h at 37°C in 100 mM sodium cacodylate buffer with 1% paraformaldehyde and 0.025% glutaraldehyde. Fixed cells were dehydrated stepwise at increasing ethanol concentrations and embedded in LR-White with accelerator (London Resin Company, London, UK). Ultrathin sections were prepared on an Reichert Ultracut E (Leica, Wetzlar, Germany) and placed on 200-mesh Pioloform-coated Ni grids.

The sections were then incubated with anti-P46 antiserum (1:1000) or naive mouse serum (1:1000) for 1 h at 37°C and then overnight at 4°C. The sections were then treated for 1 h each with rabbit anti-mouse antibody (1:1000, DAKO) and with protein A-gold (10 nm, 1:70, Department. of Cell Biology, School of Medicine, University of Utrecht, Netherlands). Staining of subcellular structures was performed in 1% UO2 in water and Pb citrate (Reynolds, 1963). Electron micrographs were taken on a Tecnai Spirit TEM (FEI, Eindhoven, Netherlands).

Animal care and experimentation were performed in accordance with the German Federal Animal Protection Laws, in particular §§4, 7 and 10a, in the animal facility of the Bernhard Nocht Institute for Tropical Medicine (Hamburg, Germany).

### Statistical and sequence analysis

Significance was assessed by *U*-test (Mann and Whitney, 1947). For macrophage infection rates, the paired Student's *t*-test was applied to account for different macrophage cell preparations. All statistical analyses were performed using the Prism Software (GraphPad). Sequence analysis was performed by a commercial provider (AGOWA, Berlin). We used the MacVector™ suite software (Version 10.6) for *in silico* sequence analysis. BLAST searches were performed at standard settings, using the GeneDB website (http://tritrypdb.org/tritrypdb/). Open reading frames were identified both by on-site analysis, using MacVector™, and by mining of the *L. major* and *L. infantum* Genome Databases.
